# Gold Nanostar Colorimetric Detection of Fructosyl Valine as a Potential Future Point of Care Biosensor Candidate for Glycated Haemoglobin Detection

**DOI:** 10.3390/bios9030100

**Published:** 2019-08-14

**Authors:** Danielle Wingrove Mulder, Masauso Moses Phiri, Barend Christiaan Vorster

**Affiliations:** Center for Human Metabolomics, North-West University, Hoffman street, Potchefstroom 2520, South Africa

**Keywords:** biosensor, colorimetric, diabetes mellitus, gold nanostars, glycated haemoglobin and hydrogen peroxide

## Abstract

Diabetes Mellitus is a growing global concern. The current methods used to detect glycated haemoglobin are precise, however, utilise expensive equipment, reagents and consumables. These are luxuries which rural communities cannot access. The nanotechnology methods which have been developed for glycated haemoglobin detection are predominantly electrochemically based, have complicated lengthy fabrication processes and utilise toxic chemicals. Here a fructosyl amino acid oxidase gold nanostar biosensor has been developed as a potential future point of care biosensor candidate for glycated haemoglobin detection. The workup done on this biosensor showed that it was able to give a spectrophotometric readout and colorimetric result with naked eye detection in blank serum spiked with fructosyl valine.

## 1. Introduction

The growing prevalence of Diabetes Mellitus is a global concern and South Africa is no exception [1]. Glycated haemoglobin (HbA_1C_) is one of the three confirmatory diagnostic tests for diabetes along with fasting plasma glucose and oral glucose tolerance test [2,3]. HbA_1C_ is haemoglobin HbA that has glucose covalently attached to the N-terminal valine of the beta chain via a nonenzymatic attachment process [4]. HbA_1C_ concentration is dependent on the erythrocyte life span and the glucose concentration of glucose found in the blood, therefore, any condition which influences the 120 day erythrocyte life span will also affect HbA_1C_ [4]. HbA_1C_ is not only used in diabetes diagnosis but is also a recognised risk factor for cardiovascular disease [5,6]. In addition to its diagnostic application HbA_1C_ is used also to monitor glycemic control, evaluate the need for therapy change and predict the development of microvascular complications.

There are more than 150 described methods available for HbA_1C_ determination. Most of these can be classified as high-performance liquid chromatography (HPLC), immunoassays, affinity chromatography, capillary electrophoresis and enzymatic assays [4]. Selection of the most appropriate method given the clinical requirement in a specific setting is a trade-off between specificity given the haemoglobin variants and genetic variation, speed of analysis and skills required, and lastly infrastructure requirement and cost of testing [2,3,7,8,9,10,11,12]. Currently there are also nanotechnology based assays for HbA_1C_ determination, however, most of these methods utilise electrochemistry which have lengthy complicated fabrication processes, use toxic chemicals and may have glucose interference [13,14,15,16,17].

The enzymatic assay utilises a *Bacillus* sp. protease in a proteolytic digestion to release glycated valine from haemoglobin beta chains. The glycated valine then serves as a substrate for fructosyl amino acid oxidase (FAO) which produces hydrogen peroxide as a product [11]. By using the recombinant fructosyl valine oxidase isolated from *Escherichia coli* species it was shown that there was no interference from glucose, bilirubin, uric acid, triglycerides etc. as it had an increased specificity for glycated valine [11,18]. Detection is then achieved by the addition of horseradish peroxidase and a chromatogen.

**Figure 1:**
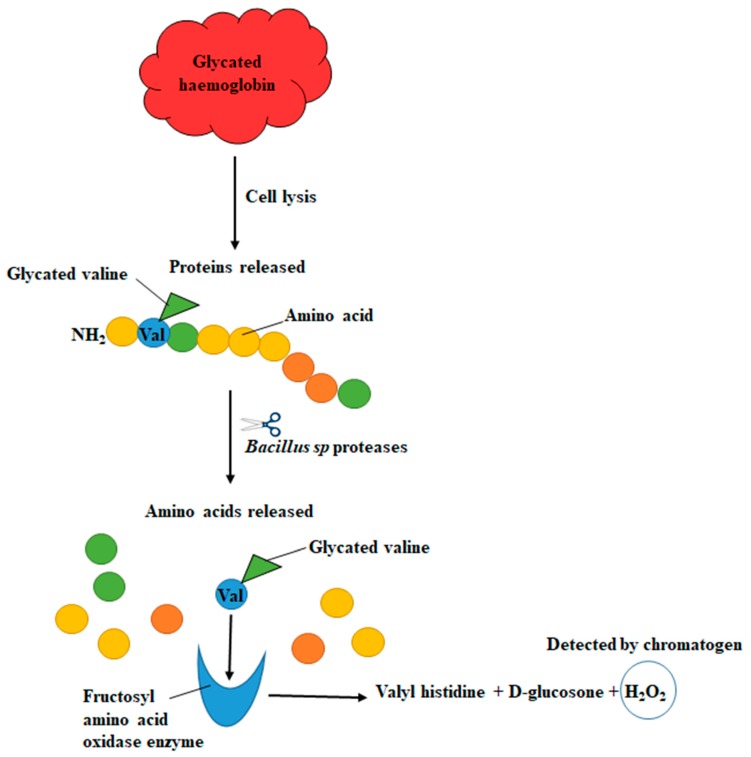
Illustration of the enzyme assay used to measure HbA_1C_ [11,19].

The reaction scheme for this reaction is illustrated in Figure 1.

Despite the niftiness of the method the assay, it is expensive which limits widespread implementation and POC testing (Figure 1).

Advances in nanotechnology have resulted in a wide range of nanosensing platforms for POC testing which have unique properties that are revolutionising the medical sector, particularly those comprised of gold nanoparticles [20]. Their easy synthesis method and their unique physical properties is what makes gold nanoparticles useful. This allows for manipulation and design [21]. Functional gold nanoparticles have been successfully used in protein, enzyme, oligonucleotide, metal ions and small molecule detection and can act as both the molecular acceptor as well as the signal transducer enhancing the sensitivity of a sensor [22,23]. As an added benefit functionalising gold nanoparticles with enzymes has been shown to stabilise both the gold nanoparticle and maintain the enzyme performance especially in a complex biological matrix [24,25]. Gold nanostars are particularly useful as biosensor detectors due to the fact that the protruding arms add to the lightning rod effect and plasmonic contributions [26]. A change in the absorption spectrum is associated with the gold nanostars surface electrons oscillations. The oscillations are influenced by the gold nanostars size, shape, arm density, surface coating and dielectric environment and can be detected by changes in scattering or absorption spectra or colour variation of the nanoparticle solution [27,28,29,30]. In literature it was shown that the localised surface plasmon resonance (LSPR) of gold nanostars shifts in response to silver ions which are reduced on to the nanostars surface in the presence of hydrogen peroxide [31].

The difference between traditional organic dye probe reactions and gold nanoparticle-based colorimetric assays is the low extinction coefficients of organic dyes rendering them less sensitive than the high gold nanoparticle extinction coefficients owed to their LSPRs. This has resulted in the gold nanoparticle-based colorimetric assays having a detection limit in the nanomolar range and in some instances lower [30]. Despite the impressive level of detections and signal amplifications of the nanoparticles they also have their challenges. These challenges include interferences in biological environments, long incubation times as well as complicated fabrication processes [32].

The aim of this research was to develop and adapt the enzymatic HbA_1C_ assay by utilising a gold nanostar biosensor as opposed to a traditional chromatogen. Such a modification will enable smaller sample volumes and quantification based on colour changes which are considered favourable for future POC applications. The results obtained showed functionalised gold nanostars with fructosyl amino acid oxidase to be a good biosensor candidate as it produced a good colorimetric differentiation between different concentrations of fructosyl valine which was detectable by the naked eye and spectrophotometrically.

## 2. Materials and Methods

### 2.1. Materials

All syntheses were done in 5mL screw cap tubes (Ascendis Medical, Johannesburg, South Africa). The stoppered polystyrene cuvettes used for dynamic light scattering was purchased from Sigma-Aldrich. The colorimetric assays were carried out in a clear, flat bottomed 96-well plate (Corning, Corning, NY, USA). The chemicals used were purchased from Sigma-Aldrich unless stated otherwise. The chemicals used were; 4-(2-hydroxyethyl)-1-piperazineethanesulfonic acid (HEPES), gold (III) chloride trihydrate, silver nitrate (AgNO_3_), polyvinylpyrrolidone (PVP) 10,000, tris-acetate EDTA buffer (TAE), tris-EDTA buffer (TRIS), sodium hydroxide, sodium chloride, 3,3′-dithiobis(sulfosuccinimidyl propionate) (DTSSP) recombinant fructosyl amino acid oxidase isolated from *Escherichia coli* species (FAO), fructosyl valine (Industrial Analytical, Johannesburg, South Africa), glucose (Dischem, Cape Town, South Africa) and Medidrug Basis-line S human blank serum (Industrial Analytical, Johannesburg, South Africa).

### 2.2. Instrumentation 

All spectra for the research were obtained using a HT Synergy microplate spectrophotometer scanned at 400–800 nm wavelengths and Gen5.1 as the corresponding software (BioTEK). Dynamic light scattering (DLS) was used to estimate the hydrodynamic diameter of the nanostars functionalised with fructosyl amino acid oxidase [33,34]. DLS was performed on a Zetasizer Nano (Malvern, Royston, UK) in backscatter mode using Zietasizer version 6.20 software and stoppered polystyrene cuvettes limiting dust contamination. The morphology of the functionalised nanostars were determined with high resolution transmission electron microscopy (HR-TEM) and energy-dispersive X-ray spectroscopy (EDS) on a Tecnai F20 high resolution transmission electron microscope. The samples were prepared by spotting the nanostars on to copper grids and air drying them. The characterisation of the functionalised nanostars were determined in 75% agarose gel electrophoresis in 1 × TAE, 50 V for 45 min. The samples were prepared by adding 8 µL nanostars to 4 µL 50% glucose solution [35].

### 2.3. Nanostar (AuNs) Synthesis 

The gold nanostars were synthesised using the HEPES buffer method described and characterised by our laboratory [36]. Briefly, 2 mL 100 mM HEPES buffer (pH 7.4) was added to 3 mL deionised water (Millipore, 18.2 ΩM), followed by 20 µL 50 mM gold (III) chloride trihydrate (HAuCl_4_·4H_2_O) and 4 µL 1 mM silver nitrate. The screw cap tube was mixed by end-to-end tube inversion and left to stand at room temperature for approximately 30 min until the solution turned a blue colour. The nanostars were then capped with 600 µL 25 mM PVP, the tube was inverted a few times and left to stand at room temperature overnight. The samples were then centrifuged and resuspended in 500 µL of deionised water after removal of the supernatant.

### 2.4. Fructosyl Amino Acid Oxidase (FAO) Functionalised Nanoparticles (FAO-AuNs) 

The nanostars were synthesised as described above (AuNs synthesis method) with the following modifications that were previously described for functionalisation with glucose oxidase [37]: Once the nanostars were synthesised by the AuNs method. Capping was performed with a PVP solution diluted to 2.5 mM and the incubation time was shortened to 90 min. The suspension was centrifuged at 1990 × g for 40 min and the FAO-AuNs were resuspended in 500 µL 100 mM HEPES (pH 6.9) after careful removal of the supernatant. Four replicates of the resuspended nanostars were then pooled to make up 2 mL. 100 µL 0.5 mM freshly made up DTSSP was added to the 2 mL stars, mixed by end-to-end inversion and left to incubate at room temperature for 3 h. Following the incubation step, 150 µL 2mg/mL FAO was added to the DTSSP functionalised nanostars and left to incubate in the fridge overnight. The following day the samples were centrifuged at 1990 × g for 40 min and the pellet was resuspended in 1 mL deionised water. The centrifugation process was repeated twice removing the excess HEPES and FAO in order to maintain star integrity [38]. Successful functionalisation was confirmed by observing the expected red shift in the UV-Vis spectrum, a decrease cathodal migration with peak broadening during agarose gel electrophoresis and an increase in hydrodynamic diameter as measured by DLS. These criteria for successful functionalisation was previously established in our laboratory along with definitive confirmation by NMR characterisation [37].

### 2.5. Feasibility Assay (unattached FAO)

Assays were carried out in a 96-well plate format. Reagents were added and pipette mixed in the following order: Ultrapure water to a final volume of 200 µL, 20 µL 10 mM TRIS buffer (pH 6.1), 15 µL AuNs, 0–0.03 mM range fructosyl valine (which is a 1:500 dilution of the expected concentration in blood samples (approximately 3–15 mM glycated haemoglobin [8,39]) and 2 µL 2 mg/mL fructosyl amino acid oxidase. The plate was then incubated on the microplate spectrophotometer for 60 min at 37 °C after which the detection solution was added using a multichannel pipette. The detection solution consisted of 2 µL 10 mM silver nitrate followed by 7 µL 500 mM sodium hydroxide. The plate was then left in the spectrophotometer for 5 min after which the spectra were obtained and the colorimetric result was observed. We have previously shown that AuNs gave a sufficient change in colour based on varying concentrations of neat hydrogen peroxide and that sodium hydroxide was the base to use in the detection solution [37].

### 2.6. Stability Assay

The stability of the FAO-AuNs were assessed with the standard sodium chloride flocculation assay (300 mM NaCl) and in blank serum. Serum was used to assess the ability of the biosensor in a complex matrix and was considered to be a reasonable proxy for haemolysates. 50 µL FAO-AuNs was added to 50 µL of NaCl and serum respectively and were read on the microplate spectrophotometer every 15 min for 2 h and again at 18 h. Since the FAO-AuNs is being utilised as a biosensor it is not necessary for the particles to be stable in the serum environment over a long period of time. A red UV-Vis spectral shift as well as broadening of the spectral curve were considered indicative of instability or the development of a protein corona [31,40].

### 2.7. Specificity Assay

Non-specific reactions by assay components were tested by omitting individual components in turn and comparing the results to a control containing all components. Fructosyl valine was replaced with 1 mM L-valine. The reagents were added and pipette mixed in the following order: Ultrapure water to a final volume of 200 µL including the detection solution, 20 µL 10 mM TRIS buffer (pH 6.1), 15 µL FAO-AuNs, 10 µL 1 mM fructosyl valine. Incubation, detection and spectral acquisitions was as described for the fructosyl valine feasibility assay with the exception that the sodium hydroxide volume was increased to 20 µL. Samples were then transferred to 1.5 mL microtubes for better colorimetric visualisation.

### 2.8. FAO-AuNs Colorimetric Assay 

The colorimetric ability of the FAO-AuNs nanostars were assessed in both water and serum each test was run in triplicate. The use of a complex, protein containing matrix is important as it is known that a protein corona forms around nanostars which can prevent colour development [41]. Both assays were optimised chemometrically. The serum samples were prepared by spiking blank serum with 12 mM fructosyl valine followed by dilution in water to range 0.3–1.5 mM. In water a 0.6 to 3 mM range was optimal. The assay procedure was as described for the FAO specificity assay accept for adding 2 µL fructosyl valine range of concentrations and 20 µL and 25 µL 500 mM sodium hydroxide to the water and serum assay respectively. Results were observed after approximately 10 min incubation with the detection solution. The final fructosyl valine concentration was a 1000× dilution of the expected 3 mM to 15 mM HbA reference range.

## 3. Results and Discussion

**Figure 2:**
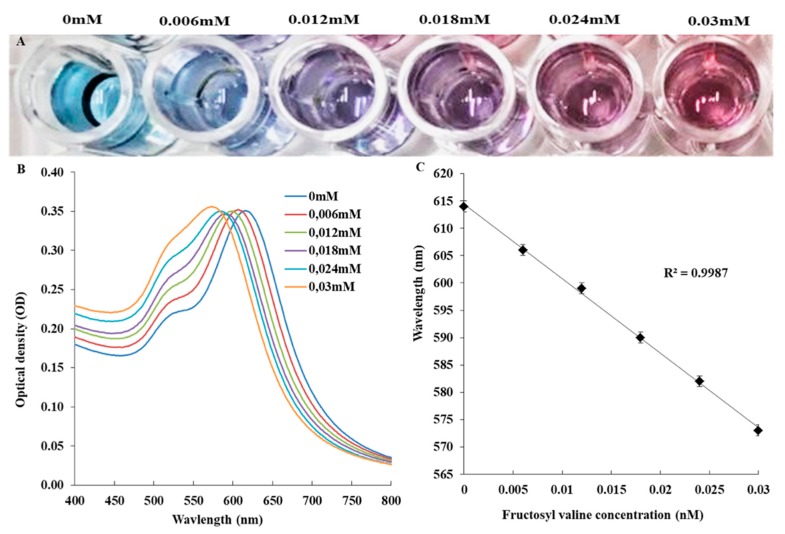
Feasibility assay. (**A**) Photograph showing the colour change of the nanostar with increased fructosyl valine concentration. (**B**) The corresponding UV-vis spectra and (**C**) scatter plot of the wavelength at ODmax and fructosyl valine concentration with linear regression fit.

The results for the feasibility assay are presented in Figure 2. An increase in the fructosyl valine concentration resulted in the colour of the solution changing from blue, to purple to red and a blue shift (to the left) in the spectral curve. The change in the wavelength at ODmax was linearly correlated with the fructosyl valine concentration over the tested range. This indicated that the FAO reaction produced sufficient hydrogen peroxide in correlation with the concentration of substrate added rendering this a feasible option for HbA_1c_ measurement.

**Figure 3:**
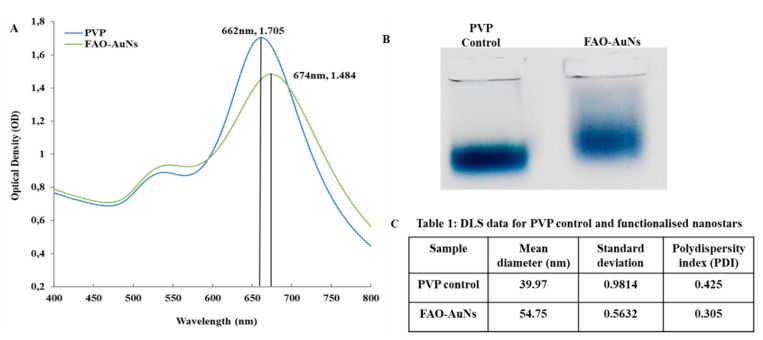
Confirmation of FAO-AuNs functionalisation. (**A**) UV-Vis spectra, (**B**) agarose gel electrophoresis and (**C**) DLS results of functionalised and unfunctionalised nanostars.

Evidence for successful FAO-AuNs functionalisation is presented in Figure 3. The 12 nm red shift with peak broadening, the decreased cathodal agarose gel electrophoresis migration pattern and 14.78 nm increase in the hydrodynamic diameter of the nanostars after functionalisation were consistent with our previous findings on glucose oxidase functionalisation as well as with that shown by Xi et. al. This is, therefore, indicative of successful functionalisation [37,42].

**Figure 4:**
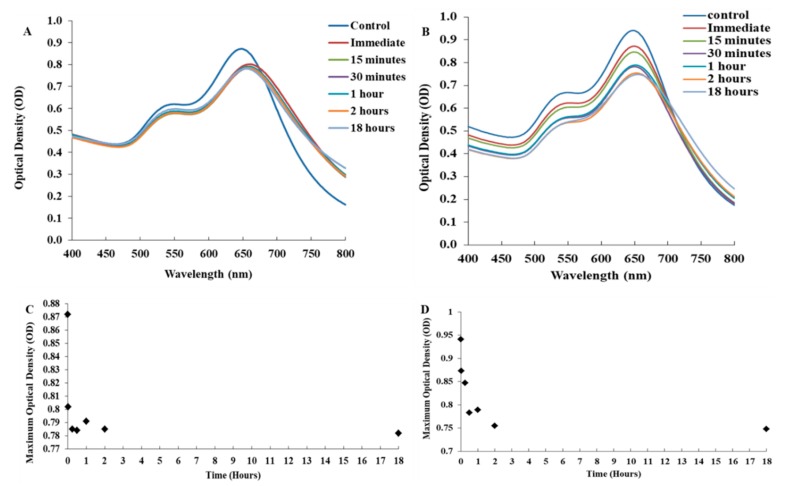
Stability of the FAO-AuNs in serum (**A**,**C**) and salt (**B**,**D**).

The stability assay data is shown in Figure 4. The FAO-AuNs were stable in the serum environment as the initial response of the nanostars resulted in a sudden shift of the spectrum and then maintained the same characteristic as the control over time (Figure 4B). In the salt environment the maximum optical density (OD max) declined by 30% within the first 30 min with a marginal red shift after which it remained stable for at least up to two hours (Figure 4A). The biosensor did not need to be stable in a complex matrix such as serum for long periods as the result should be obtained in the shortest possible time frame. There was a horizontal relationship between the maximum optical density and time for the serum environments indicating nanostar stability (Figure 4C). The maximum optical density and time for the salt environment showed a decline in relationship for the first 2 h and then plateaued out until the 18 h time point (Figure 4D).

**Figure 5:**
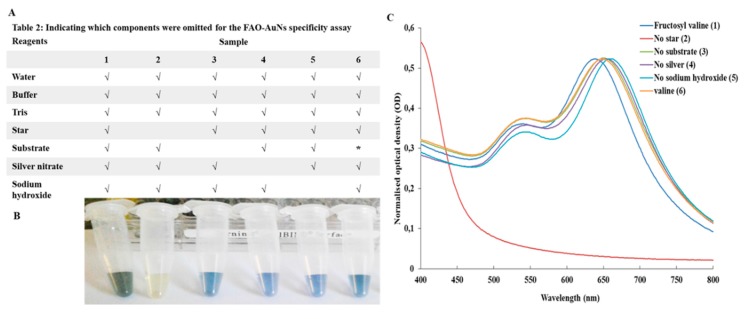
**Specificity assay**. (**A**) Table showing omitted reaction components accept for * which indicates the replacement of fructosyl valine with L-valine. (**B**) Photograph of the colours obtained for the reaction as laid out in the table. (**C**) Corresponding normalised UV-Vis spectra.

Data supporting the specificity of the FAO-AuNs assay is presented in Figure 5. Although marginal shifts did occur with the omission of various reaction components, the magnitude was less than the shift with all components present even without prior optimisation of the reaction conditions. In addition silver reduction without the presence of nanostars did not result in a significant shift.

**Figure 6:**
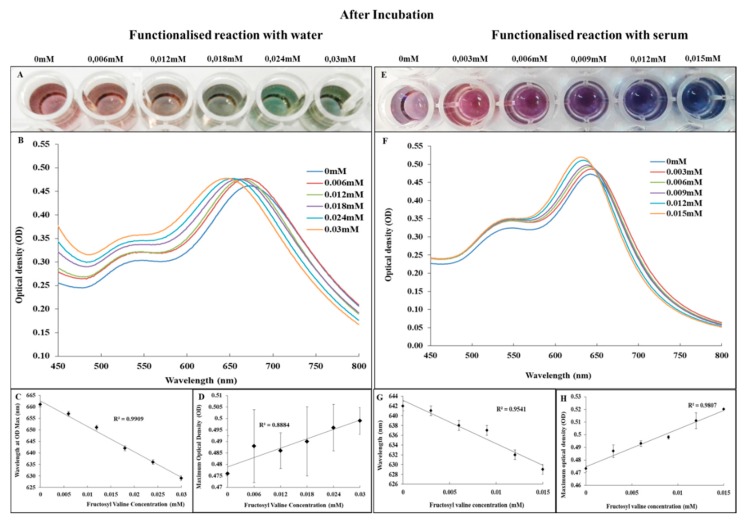
Matrix effects evaluation assays: (**A**) Photograph showing the colour of the FAO-AuNs in water. (**B**) UV-Vis spectra for FAO-AuNs in water immediately after detection solution is added. (**C**,**D**) Linear relationships for FAO-AuNs (in water) between fructosyl valine concentration and shift in wavelength and maximum optical density respectively. (**E**) Photograph showing the colour of the FAO-AuNs in serum after incubation. (**F**) UV-Vis spectra for FAO-AuNs in serum immediately after addition of detection solution. (**G**,**H**) Linear relationships for FAO-AuNs (in serum) between fructosyl valine concentration and wavelength and maximum optical density respectively.

The compound figure in Figure 6, shows the contrast of colour obtained between the water and serum environment as well as the spectral and linearity data.

**Figure 7:**
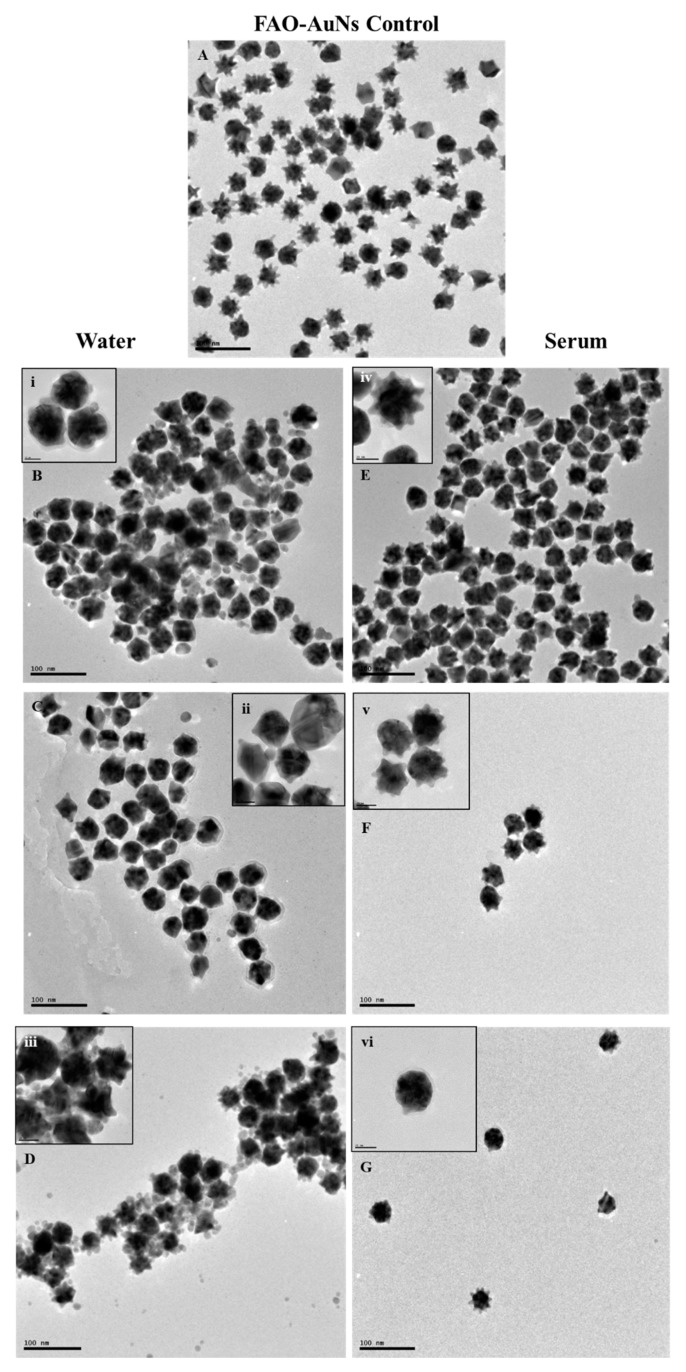
TEM images showing the morphological change of the FAO-AuNs in water and serum for varied fructosyl valine concentrations. (**A**) Is the morphology of FAO-AuNs after functionalisation. In the water environment (**B**) 0 mM, (**C**) 0.018 mM and (**D**) 0.03 mM. In the serum environment (**E**) 0 mM, (**F**) 0.009 mM and (**G**) 0.015 mM respectively. The inserts for each image shows a closer view of the particle morphology.

In Figure 6A, it can be seen that the FAO-AuNs solution changed to a colour range of red, orange, yellow and green. The spectral shift in Figure 6B was a blue shift with the highest concentration of fructosyl valine blue shifting the most which concurred with Figure 2B. This pattern in spectral shift and constant OD max was described by Zang et.al as a change in morphology by etching and growth while maintaining the nanoparticle size as no change in OD max was noted [30]. This was confirmed by the images in Figure 7B–D. The colour change difference between Figure 6A,E could be attributed to a protein corona which forms around the nanostars changing the nanostar physiochemical properties [41]. The FAO-AuNs in serum (Figure 6F), also showed a blue shift with the highest concentration of fructosyl valine shifting the most albeit with some change in OD max.

**Figure 8:**
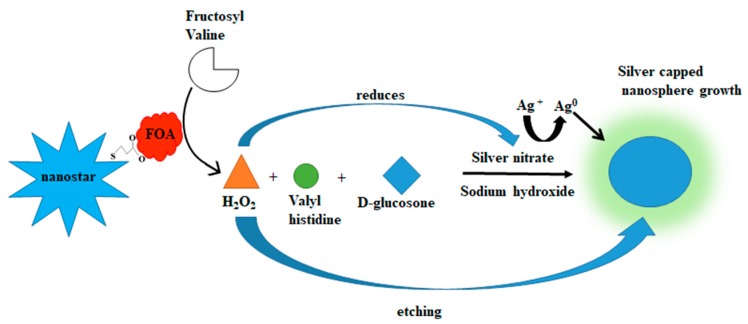
Proposed reaction scheme for the colorimetric reaction obtained by the FAO-AuNs in serum.

The possible mechanism contributing to the colour change was investigated by viewing the particle morphology via TEM. It is proposed that the colour was attributed via enzyme-mediated etching and growth of the gold nanostars [30]. A proposed scheme is illustrated in Figure 8 [43].

The TEM images of the nanoparticles in both the water and serum are shown in Figure 7 below.

When the nanostar was functionalized, the shape of the nanostar was maintained as seen in the TEM image in Figure 7A. The TEM images seen in Figure 7E–G support Figure 8. The morphology change of the FAO-AuNs in serum as seen in Figure 7E–G resulted in the shortening of the FAO-AuNs arms compared with the functionalisation control (7A). The arms were gradually removed until finally resulting in more spherical nanoparticles. The etching and growth of the FAO-AuNs in water followed a different pattern where the shape started off more spherical and as the substrate concentration increased the shape became more polyhedral in nature with a final poly-morphological sample coated by silver nanoparticles [37].

As seen in Figure 6 and Figure 7, the biosensor was able to detect low levels of fructosyl valine which was via the etching and growth of the gold nanostar. The combination of the TEM images and the colorimetric data gave an indication to the mechanism attributing to the colour change. This gives a better understanding of how these particular nanostars react in a complex matrix such a serum which will assist with optimisation when applying these biosensors to blood.

## 4. Conclusions

It was previously shown that using silver as a shape directing agent in a HEPES seedless synthesis method that more homogenous gold nanostars with multiple branches were produced [36]. These nanostars were functionalised with glucose oxidase as a model enzyme which showed to be a promising biosensor scaffold for the detection of hydrogen peroxide generated by an enzyme-substrate reaction [37]. This model was then generalised to another hydrogen producing enzyme such as fructosyl valine for HbA_1c_ determination. When compared to traditional chromatogens, functionalised FAO-AuNs offered the advantage of colorimetric naked eye and spectrophotometric detection by using smaller sample volumes which are desirable for POC applications. For potential future application, the quantification of the colour changes obtained could possibly be done via smartphone apps or an external mobile add-on [44,45,46,47]. The biosensor was also stable in a complex matrix such as serum. Future work in our laboratory will, therefore, be in optimising the blood sample preparation by cleaving the glycated valine from haemoglobin with limited reagents as to limit interference for the colorimetric assay platform which was established in this study. The future work will also include validation using patient samples and comparing it to the traditional enzyme method for which ethics will be required.
